# Induction of Apoptosis and Decrease of Autophagy in Colon Cancer Cells by an Extract of Lyophilized Mango Pulp

**DOI:** 10.3390/ijerph20054165

**Published:** 2023-02-25

**Authors:** Gustavo Argenor Lozano-Casabianca, Sandra Sulay Arango-Varela, María Elena Maldonado-Celis

**Affiliations:** 1 School of Nutrition and Dietetics, University of Antioquia, Medellín 050010, Colombia; 2 Facultad de Ciencias Exactas y Aplicadas, Instituto Tecnológico Metropolitano (ITM), Institución Universitaria, Medellín 050034, Colombia

**Keywords:** *Mangifera indica* L., colorectal cancer, apoptosis, autophagy, matrix metalloproteinases

## Abstract

Previous studies have indicated that mango fruit has a chemopreventive capacity against colorectal cancer cells. The objective of this research was to evaluate the effect of an aqueous extract of lyophilized mango pulp (LMPE) on colon adenocarcinoma cells (SW480) and their metastatic derivatives (SW620) death and cellular invasion. DNA fragmentation was assessed by TUNEL assay; autophagy and expression of DR4 and Bcl-2 by flow cytometry; the expression of 35 apoptosis-related proteins and of matrix metalloproteinases 7 and 9 by immunodetection; and the invasive capacity of the cells by Boyden chamber. The results showed that LMPE at 30 mg/mL and 48 h of exposure results in DNA fragmentation and apoptosis in SW480 (*p* < 0.001) and SW620 (*p* < 0.01) cells. Additionally, LMPE decreased autophagy in the SW480 and SW620 cell lines (*p* < 0.001), which could sensitize them to the DNA damage generated by LMPE. The LMPE did not modulate the expression of matrix metalloproteinases 7 and 9, nor did it affect cellular invasion processes in the SW480 and SW620 cell lines. In conclusion, LMPE induces apoptosis and decreases autophagy in SW480 and SW620 cells.

## 1. Introduction

Colorectal cancer (CRC) global incidence and mortality rates are increasing dramatically [1]. According to the projection of aging, population growth, and human development, the overall number of new CRC cases will reach 3.2 million by 2040. Increased exposure to environmental risk factors caused by lifestyle changes and a westernized diet is primarily responsible for the increased incidence of CRC [2]. Once CRC has been diagnosed and staged, its treatment may include but is not limited to, surgery, radiation therapy, immunotherapy, and chemotherapy. However, the unwanted side effects of the treatment and the recurrence promote the search for alternatives for its treatment [3]. Dietary bioactive compounds from fruits and vegetables have been reported to have a biological effect on CRC [3], and this has served in the search for less toxic treatment alternatives.

One of the most widely consumed fruits worldwide is the mango (*Mangifera indica* L.) [4], and it is considered a functional food due to its content of bioactive compounds [5]. Studies in vitro and murine models have shown its chemopreventive capacity against different types of cancer [6], including CRC [7,8,9,10].

Our group evidenced that an aqueous extract of lyophilized mango pulp (LMPE) is a source of bioactive compounds with cytotoxic capacity against colon adenocarcinoma cells (SW480) and their metastatic derivatives (SW620); this effect was selective with respect to non-malignant skin fibroblastic cells. The mechanisms of action of the extract involved in its cytotoxic and antiproliferative activity were an increase in oxidative stress, arrest of the cell cycle, and induction of apoptosis [11].

Along with apoptosis, autophagy, and cellular invasion processes are considered therapeutic targets of natural agents with chemopreventive properties [12]. Apoptotic processes regulate cell survival and death in a coordinated manner. However, during the development of cancer, this mechanism can be altered, thus favoring the progression of the tumor and resistance to the usual anti-tumor therapies [13]. There are several promising new anticancer treatments derived from plant compounds that act by activating the apoptotic pathway [14]. Meanwhile, autophagy—a mainly adaptive process of cells with cytoprotective function and survival in conditions of lack of nutrients and ATP deficiency [15]—occurs in numerous types of cancer, including CRC, and could be quite helpful in both its prevention and treatment [16]. Regarding invasion, this is a characteristic of cancer cells that allows them to spread to other layers of the same organ prior to migration. In the invasion process, matrix metalloproteinase (MMPs) enzymes reshape and degrade the extracellular matrix (ECM) [17]. The association of MMPs with cancer invasion and metastasis has aroused great interest in aiming to develop new antimetastatic drugs intended to inhibit the activity of these enzymes [18].

The colon cancer cell lines SW480 and its metastatic derived cells SW620 constitute an in vitro model of cancer development. A 50-year-old male patient provided the SW480 cell line (Dukes stage B), while the SW620 cell line (Dukes stage C) was derived from a lymph node metastasis from the same patient [19].

Therefore, the objective of this research was to evaluate the effect of an aqueous extract of lyophilized mango pulp (LMPE) on the death and invasion processes of colon adenocarcinoma cells (SW480) and their metastatic-derived cells (SW620). The research results led to the conclusion that LMPE induced apoptosis and decreased autophagy in SW480 and SW620 cells. Furthermore, LMPE did not affect cell invasion processes in these cell lines.

## 2. Materials and Methods

### 2.1. Preparation of Extract of Lyophilized Mango Pulp (LMPE)

A commercial lyophilisate of mango cv. Yulima pulp was used (Fontus SAS, Bogotá, Colombia). The lyophilisate was dissolved in sterile distilled water for 24 h at 37 °C with continuous stirring at 180 rpm. The mixture was filtered with sterile gauze and lyophilized again in a vacuum chamber at −50 °C and a pressure of 0.427 ± 0.5 mm Hg (LyoQuest, Telstar^®^, Terrassa, Spain). The lyophilized extract was stored at −20 °C and protected from light until use.

### 2.2. Maintenance and Treatment of Cells

The SW480 and SW620 cell lines were obtained from the European Collection of Animal Cell Culture (ECACC, Salisbury, UK). Cells were cultured following the protocol proposed by Maldonado et al. [20]. Dulbecco’s Modified Eagle Medium (DMEM) supplemented with 25 mM glucose, 2 mM L-glutamine, 10% fetal bovine serum, 100 U/mL penicillin, 100 μg/mL streptomycin, and 1% non-essential amino acids (Invitrogen, Carlsbad, CA, USA) was employed. Incubations were performed at 37 °C and 5% CO_2_. A solution of 100 mg LMPE/mL in 3% DMEM (DMEM supplemented with 25 mM glucose, 2 mM L-glutamine, 3% fetal bovine serum, 100 U/mL penicillin, 100 μg/mL streptomycin, 1% non-essential amino acids, 10 μg/mL insulin, 5 μg/mL transferrin, 5 ng/mL selenium) was prepared. The mixture was sonicated for 20 min at 42 kHz, then centrifuged at 5000 rpm for 20 min, and the supernatant was collected and filtered on a 0.2 µm membrane. Finally, it was stored in darkness at −20 °C until use. Cells were treated with different concentrations of LMPE (10, 20, and 30 mg/mL), and untreated cells (0 mg LMPE/mL) were used as a control.

### 2.3. Terminal Deoxynucleotidyl Transferase (TdT)-Mediated dUTP Nick-End Labeling (TUNEL) Assay

The fragmentation of cellular DNA as a late marker of apoptosis was determined by the TUNEL method [21]. 1 × 10^6^ cells/well were maintained in 6-well dishes at 37 °C with 5% CO_2_ for 24 h, then treated for 48 h with the extract at 30 mg/mL. Cells were collected with trypsin and washed with PBS [20]. APO-DIRECT™ detection kit (Catalog #APT110, Chemicon^®^ International, Inc., Temecula, CA, USA) was used following the manufacturer’s instructions. This kit contains apoptotic cells (positive control cells) and non-apoptotic cells (negative control cells). Data from at least 10,000 events per sample were collected by flow cytometry (FACSCanto^TM^ II, BD Biosciences, Franklin Lakes, NJ, USA) and analyzed using the FlowJo™ software (version 7.6.2, Becton, Dickinson and Company, Ashland, OR, USA).

### 2.4. Change in Mitochondrial Membrane Potential (ΔΨm)

To determine changes in the mitochondrial membrane potential (ΔΨ*m*) due to the effect of the LMPE, the dye DiOC_6_ (3,3’-dihexyloxacarbocyanine iodide) [20] was used. 1 × 10^6^ cells/well were seeded in 6-well dishes and treated as described in the TUNEL assay. Cells were collected with trypsin, washed, and resuspended in PBS, and incubated with DiOC_6_ (Thermo Fisher Scientific, Waltham, MA, USA) at a final concentration of 700 nM at 37 °C for 30 min. Data from at least 10,000 events per sample were collected by flow cytometry and analyzed using FlowJo™.

### 2.5. Detection of TRAIL-R1 (DR4) Death Receptor Expression

1 × 10^6^ cells/culture well were seeded in 6-well dishes and treated as described in the TUNEL assay. Cells were collected with trypsin, washed, and resuspended in PBS. They were then incubated with the mouse-produced anti-CD261-APC monoclonal antibody, clone DR-4-02 (1:100 dilution) (Catalog #SAB4701084, Sigma Aldrich, Saint Louis, MO, USA) for 30 min at 4 °C in the dark. After two wash steps, cells were resuspended in PBS, and data from at least 10,000 events per sample were collected by flow cytometry and analyzed with FlowJo™ [22].

### 2.6. Detection of the Bcl-2 Expression

1 × 10^6^ cells/culture well were seeded in 6-well dishes and treated as described in the TUNEL assay. Cells were harvested using trypsin, washed, and resuspended in PBS. The cells were then fixed with 70% ethanol (4 °C), washed, and resuspended in PBS. Cells were then incubated with anti-Bcl-2, mouse monoclonal, Bcl-2/100 clone (1:100 dilution) (Catalog #SAB4700861, Sigma Aldrich, Saint Louis, MO, USA) for 30 min at 4 °C in the dark. After two washes, goat anti-mouse IgG (H + L) conjugated to fluorescein (1:10 dilution) (Catalog #12-506, EMD Millipore Corporation, Temecula, CA, USA) was added for 30 min at 4 °C. After two washes, cells were resuspended in PBS and data from at least 10,000 events per sample were collected by flow cytometry and analyzed using FlowJo™ [23].

### 2.7. Protein Analysis Related to Human Apoptosis in Cell Lysates

1 × 10^7^ cells were maintained at 37 °C and 5% CO_2_ in 100 mm diameter culture dishes. After 24 h the cells were treated with the LMPE at 30 mg/mL for 48 h. Cells were collected using trypsin and washed in PBS. After removing any remaining PBS, cells were solubilized at 1 × 10^7^ cells/mL in lysis buffer 17 (Catalog #895943, R & D Systems, Minneapolis, MN, USA) supplemented with 10 µg/mL aprotinin (Catalog #4139, Tocris™, Minneapolis, MN, USA), 10 µg/mL leupeptin (Catalog #1167, Tocris™, Minneapolis, MN, USA), and 10 µg/mL pepstatin (Catalog #1190, Tocris™, Minneapolis, MN, USA). The lysates were gently stirred at 2–8 °C for 30 min and centrifuged at 14,000× *g* for 5 min. The supernatant was stored at ≤−70 °C until use. Total protein was quantified with the commercial kit for total protein quantification with Pierce™ Bicinchoninic Acid (BCA) Protein Assay Kit (Thermo Fisher Scientific). The protein samples obtained (250 µg/assay) were analyzed using the commercial Proteome Profiler Human Apoptosis Array Kit (Catalog #ARY009, R&D Systems, Minneapolis, MN, USA) following the manufacturer’s instructions. The relative expression of the proteins was determined in a ChemiDoc XRS + Cell Imaging system (BioRad, Hercules, CA, USA). The results were quantified in the Image Lab 6.1 software (BioRad, Hercules, CA, USA) and expressed as fold change versus control [24].

### 2.8. Bioinformatic Analysis of LMPE-Modulated Proteins

For the bioinformatic analysis, the STRING^®^ platform was used to predict a network of the interaction of the proteins modulated by the extract at 30 mg/mL in SW480 and SW620 cells, estimating the biological processes and the signaling pathways involved, as suggested by von Mering et al. [25].

### 2.9. Autophagy Death Determination

Autophagy was detected by a patented autophagosome fluorescent marker [26] available as a commercial autophagy kit (Catalog #MAK138, Sigma Aldrich, Saint Louis, MO, USA). 1 × 10^6^ cells/culture well were seeded in 6-well dishes and treated as described in the TUNEL assay. Cells were harvested using trypsin, washed, and resuspended in PBS. Subsequently, the commercial autophagy kit was used following the manufacturer’s instructions. In addition, 0.1 µM rapamycin was used as a positive control. Data from at least 10,000 events per sample were collected by flow cytometry and analyzed using FlowJo™.

### 2.10. Detection of MMP-7 and MMP-9 Expression

1 × 10^7^ cells were maintained at 37 °C and 5% CO_2_ in 100 mm diameter culture dishes. After incubation for 24 h, the cells were treated for 48 h with the extract at 30 mg/mL. The cells were then lysed, and the total protein was quantified using Pierce™ Bicinchoninic Acid (BCA) Protein Assay Kit (Thermo Fisher Scientific) as described above. The proteins obtained (100 μg/well) were analyzed using in vitro immunoenzyme assays for quantitative measurement of human MMP-7 (Catalog #RAB0369, Sigma Aldrich, Saint Louis, MO, USA) and human MMP-9 (Catalog #RAB0372, Sigma Aldrich, Saint Louis, MO, USA) following the manufacturer’s instructions. The absorbance of each well was determined at 450 nm in the microplate reader (Varioskan^TM^ LUX Multimode Microplate Reader, Thermo Fisher Scientific, Waltham, MA, USA). The concentration of MMP-7 and MMP-9 in the samples was calculated by comparing the absorbance of the samples with the corresponding standard curves [27].

### 2.11. Invasion Assay

An invasion assay was performed through an extracellular matrix (ECM), based on the Boyden chamber principle, using a commercial kit (Catalog #ECM555, EMD Millipore Corporation, Temecula, CA, USA) and following the manufacturer’s instructions [28]. In this trial, 20,000 cells/well in 96-well dishes were treated for 18 h with the extract at 10, 20, and 30 mg/mL. The fluorescence of each well was determined at 480/520 nm using a fluorescence plate reader (Varioskan^TM^ LUX Multimode Microplate Reader, Thermo Fisher Scientific, Waltham, MA, USA).

### 2.12. Statistical Analysis

Unless otherwise stated, the results were expressed as mean ± standard error of the mean (SEM). For the TUNEL assay and the determination of autophagy, parametric comparative statistical analyses (ANOVA) were carried out. In order to comply with the ANOVA assumptions, the TUNEL assay data were transformed to Log_10_. Differences in treatment means were analyzed with Dunnett’s post-hoc test. A student *t* test was used to compare cell groups in terms of apoptosis-related proteins, DR4, Bcl-2, MMP-7, and MMP-9. The Kruskal-Wallis non-parametric test was used to analyze the cell invasion assay. In all cases, a *p* < 0.05 was considered significant. The statistical software GraphPad Prism 8.0 (GraphPad Software Inc., San Diego, CA, USA) was used for data analysis. All assays were conducted at least in triplicate.

## 3. Results

### 3.1. Effect of LMPE on SW480 and SW620 Cell Apoptosis

**Figure 1:**
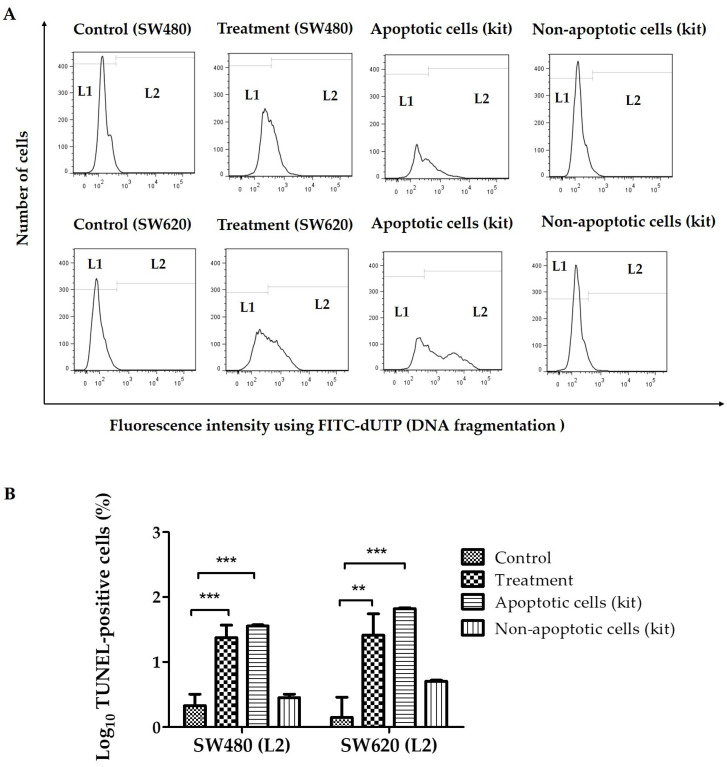
Effect of an aqueous extract of lyophilized mango pulp (LMPE) on DNA fragmentation in SW480 and SW620 cell lines. The cells were exposed to 30 mg LMPE/mL for 48 h. Untreated cells (0 mg LMPE/mL) were used as a control. The kit used has apoptotic cells (positive control) and non-apoptotic cells (negative control). Representative histograms of fluorescence intensity using fluorescein isothiocyanate (FITC)-dUTP (detected by flow cytometry) in SW480, SW620, apoptotic (positive control), and non-apoptotic (negative control) (**A**) cells. TUNEL-negative cells (with lower DNA fragmentation) are located in L1 and TUNEL-positive cells (with higher DNA fragmentation) are in L2. Log_10_ of the percentage of TUNEL-positive cells (located at L2) (**B**). Data were expressed as mean ± SEM (*n* = 3). ANOVA test was performed to compare the groups; *p* < 0.001 for both cell lines. Differences between the group mean and control group was analyzed by Dunnett’s post hoc test; significant differences were expressed as ** *p* < 0.01 and *** *p* < 0.001.

To determine the percentage of SW480 and SW620 cells with single-strand DNA breaks and to confirm the apoptotic effects of LMPE in SW480 and SW620 cells, the TUNEL method was used. Strand breaks were detected by flow cytometry with the TdT reaction in the presence of FITC-dUTP. The kit used contains apoptotic cells (positive control) and non-apoptotic cells (negative control). Figure 1A shows representative histograms of FITC-dUTP fluorescence intensity in SW480, SW620, apoptotic (positive control), and non-apoptotic (negative control) cells. TUNEL-negative cells (with lower DNA fragmentation) are located in L1 and TUNEL-positive cells (with higher DNA fragmentation) are in L2. Figure 1B shows that in both CRC cell lines, the percentage of TUNEL-positive cells (with DNA fragmentation, apoptotic) of the control cells (untreated cells, 0 mg LMPE/mL) is lower compared to the apoptotic cells of the commercial kit (positive control), and there is no significant difference with the non-apoptotic cells of the commercial kit (negative control). Likewise, Figure 1B shows an increase in the percentage of TUNEL-positive cells SW480 (*p* < 0.001) and SW620 (*p* < 0.01) treated with the extract at 30 mg/mL for 48 h compared to their corresponding control groups (0 mg LMPE/mL). These results suggest that LMPE treatment produced DNA fragmentation and apoptosis in both cell lines.

### 3.2. Change in Mitochondrial Membrane Potential (ΔΨm)

**Figure 2:**
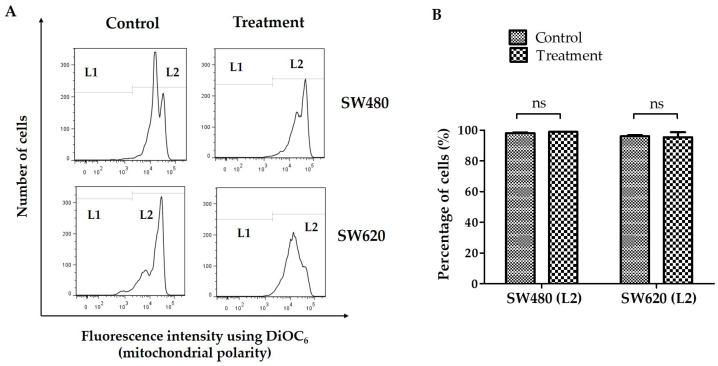
Effect of LMPE on mitochondrial membrane potential change (ΔΨ*m*) in SW480 and SW620 cell lines. Histograms represent fluorescence intensity using DiOC_6_ (detected by flow cytometry) in SW480 and SW620 (**A**) cells. The cells with the permeability of the mitochondrial membrane are located at L1 and the cells with polarized mitochondria are at L2. Percentage of SW480 and SW620 cells with the polarized mitochondrial membrane (**B**). Cells were exposed to 30 mg LMPE/mL for 48 h. Untreated cells (0 mg LMPE/mL) were used as a control. Data were expressed as mean ± SEM (*n* = 3). The differences between the control and treated cells were analyzed with Student’s *t* test, ns = non-significant difference, *p* = 0.2368 (SW480), and *p* = 0.8514 (SW620).

The fluorescent probe DiOC6 was used in flow cytometry to measure the impact of a 48-h treatment with 30 mg/mL of LMPE on the mitochondrial membrane potential. Reduced mitochondrial membrane potential is associated with a drop in the dye in the mitochondria [29]. Figure 2A shows representative histograms of fluorescence intensity using DiOC_6_ in SW480 and SW620 cells. The staining allowed the identification of the group of cells with mitochondrial membrane permeability (L1) and cells with polarized mitochondria (L2). Figure 2B shows the proportion of SW480 and SW620 cells with polarized mitochondria (L2). There was no significant difference between SW480 (*p* = 0.2368) and SW620 (*p* = 0.8514) cells treated with 30 mg LMPE/mL for 48 h compared with their corresponding control groups. That is, LMPE treatment did not cause mitochondrial membrane permeabilization in these CRC cells.

### 3.3. Detection of the Bcl-2 Protein Expression

**Figure 3:**
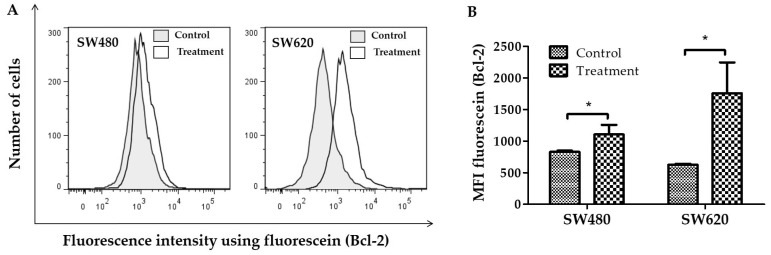
Effect of LMPE on Bcl-2 expression in SW480 and SW620 cell lines. Histograms represent fluorescence intensity using anti-Bcl-2, mouse monoclonal and goat anti-mouse IgG (H + L), fluorescein conjugate (detected by flow cytometry) in SW480 and SW620 (**A**) cells. Bcl-2 expression is indicated by the mean fluorescence intensity (MFI) of fluorescein in SW480 and SW620 (**B**) cells. Cells were exposed to 30 mg LMPE/mL for 48 h. Untreated cells (0 mg LMPE/mL) were used as a control. Data were expressed as mean ± SEM (*n* = 3). The differences between the control and treated cells were analyzed with the Student’s *t* test. The significant differences were expressed as * *p* < 0.05.

Figure 3A shows histograms representative fluorescence intensity detected by flow cytometry using anti-Bcl-2, mouse monoclonal and goat anti-mouse IgG (H+L), fluorescein conjugate in SW480 and SW620 cells. In this figure, the rightward shift of fluorescence was a sign that more cells expressed the Bcl-2 protein. By using flow cytometry, the mean fluorescence intensity (MFI) was also calculated. Figure 3B indicates that, compared to controls, the MFI of fluorescein (Bcl-2) increased in SW480 and SW620 cells (*p* < 0.05) treated with 30 mg LMPE/mL for 48 h. These results show that treatment with LMPE increased Bcl-2 expression in both cell lines.

### 3.4. Detection of DR4 Death Receptor Expression

**Figure 4:**
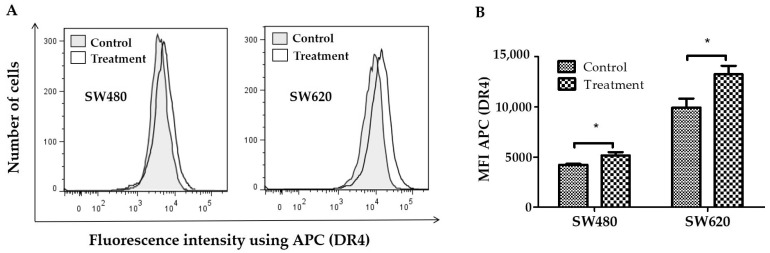
Effect of LMPE on DR4 death receptor expression in SW480 and SW620 cell lines. Histograms represent fluorescence intensity using anti-DR4-APC (detected by flow cytometry) on SW480 and SW620 cells (**A**). DR4 death receptor expression is indicated by the mean fluorescence intensity (MFI) of APC in SW480 and SW620 (**B**) cells. Cells were exposed to 30 mg LMPE/mL for 48 h. Untreated cells (0 mg LMPE/mL) were used as a control. Data were expressed as mean ± SEM (*n* = 3). The differences between the control and treated cells were analyzed with the Student’s *t* test. The significant differences were expressed as * *p* < 0.05.

In SW480 and SW620 cells, a mouse-produced anti-CD261-APC monoclonal antibody (detected by flow cytometry) was used to assess the effect of LMPE on DR4 receptor expression on the cell surface. Figure 4A shows representative histograms of fluorescence intensity using anti-CD261-APC in SW480 and SW620 cells. In this figure, the rightward shift of fluorescence was a sign that more cells expressed the DR4 receptor on their cell surface. Using flow cytometry, the MFI was also calculated. Figure 4B shows that the MFI of APC (DR4) in cells treated with 30 mg LMPE/mL for 48 h increased compared to controls (*p* < 0.05) in SW480 and SW620 cell lines. That is, treatment with LMPE increased DR4 death receptor expression in both cell lines.

### 3.5. Protein Analysis Related to Human Apoptosis in Cell Lysates

**Figure 5:**
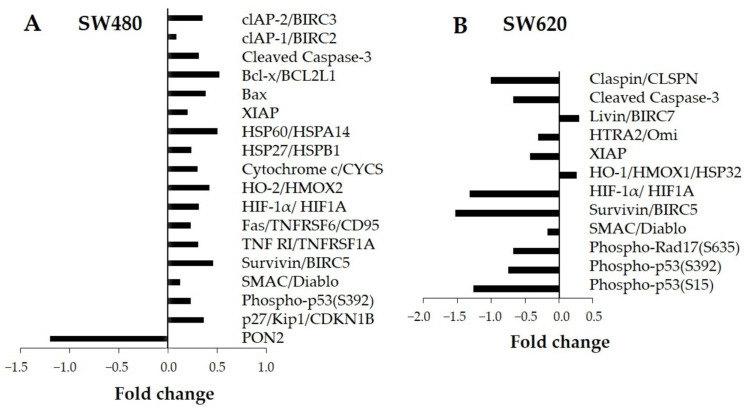
Evaluation of the effect of LMPE on modulation of apoptosis-related proteins. The protein fold changes for LMPE versus control in SW480 (**A**) and SW620 (**B**) cells. Cells were exposed to 30 mg LMPE/mL for 48 h. Untreated cells (0 mg LMPE/mL) were used as a control.

The expression of 35 apoptosis-related proteins was assessed by immunodetection. Figure 5 shows the significant values of fold change obtained from protein expression of treated cells (30 mg LMPE/mL) for 48 h versus the untreated ones (0 mg LMPE/mL) in SW480 cell lines (Figure 5A) and SW620 (Figure 5B). In SW480, the LMPE modulated the expression of 18 proteins, while in SW620, it modulated the expression of 12 proteins. In SW480, increasing levels of expression of all LMPE-modulated proteins were observed apart from PON2, the expression of which was decreased. In SW620, decreased expression of all LMPE-modulated proteins was observed except Livin and HO-1, whose showed increased expression.

### 3.6. Bioinformatic Analysis of LMPE-Modulated Proteins

**Figure 6:**
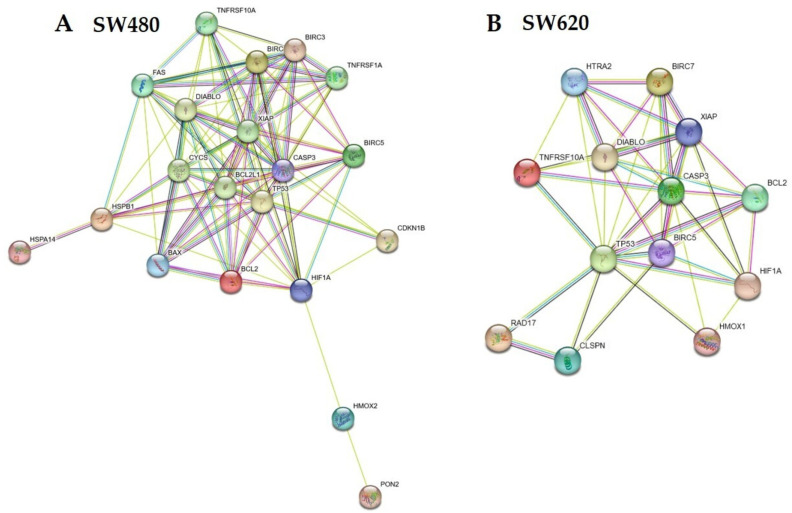
Representative network of LMPE-modulated proteins predicted by STRING^®^ for cell lines SW480 (**A**) and SW620 (**B**).

The arrangement of LMPE-modulated proteins in SW480 cells (Figure 6A) and SW620 (Figure 6B) was predicted by STRING^®^. Table 1 displays the bioinformatic analysis of the primary biological pathways and processes from the Kyoto Encyclopedia of Genes and Genomes (KEGG) after the STRING^®^ analysis of LMPE-modulated proteins. As can be seen, treatment with LMPE modulates biological processes related to apoptosis in both cell lines. That is, the analysis of the KEGG pathways confirms that the extract modulates the activation of apoptosis in both cell lines.

### 3.7. LMPE Modulated Signaling Pathways in SW480 and SW620 Cells

**Figure 7:**
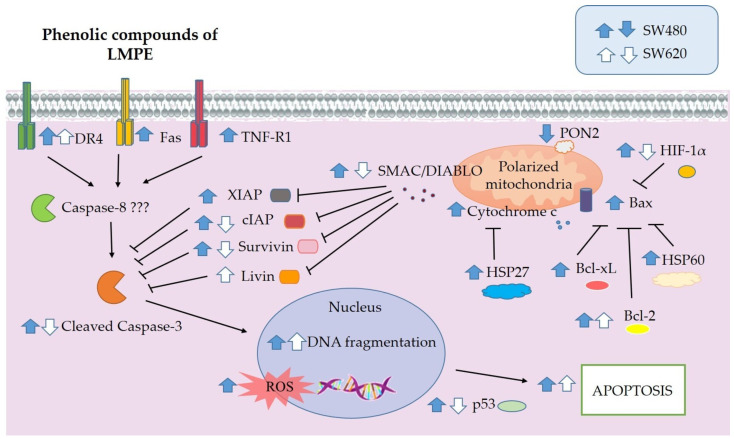
Overview of signaling pathways possibly modulated by LMPE in SW480 and SW620 cells. The blue (SW480) and white (SW620) arrows indicate the possible upward or downward regulation of the selected proteins or the studied biological processes.

From the results of the study, possible signaling pathways modulated by LMPE in SW480 and SW620 cells were inferred (Figure 7). No alteration in the mitochondrial function of SW480 and SW620 cells was found. Therefore, the extract-induced death in both cell lines may occur via apoptotic pathways without mitochondrial mediation. In SW480 cells, the extract induced increased expression of the death receptors Fas, DR4, and TNF-R1. It also upregulated the expression of cleaved caspase-3 and DNA fragmentation. When taken together, these results indicate that extract-induced apoptotic death in SW480 cells occurs extrinsically and that mitochondrial membrane permeabilization is not involved. From the results obtained in biomarkers such as DNA fragmentation and bioinformatic analysis of modulated KEGG pathways in SW620 cells treated with LMPE under the same conditions as SW480, it is possible to infer an activation of apoptosis. However, the downregulation of cleaved caspase-3 observed in SW620 raises doubts that the pathway being activated is the extrinsic pathway; therefore, it was not possible to propose the apoptotic signaling pathway triggered by the LMPE in this cell line.

### 3.8. Determination of Autophagy

**Figure 8:**
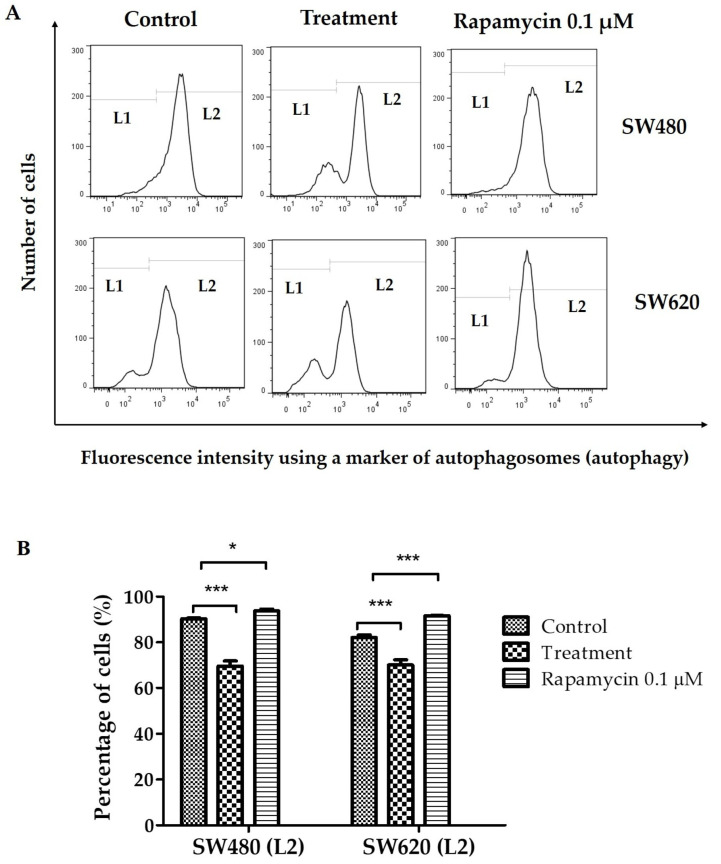
Effect of LMPE on autophagosome production (autophagy) in SW480 and SW620 cell lines. Cells were exposed to 30 mg LMPE/mL for 48 h. Untreated cells (0 mg LMPE/mL) were used as a control. 0.1 µM rapamycin was used as a positive control. Histograms represent the fluorescence intensity of an autophagosome marker (detected by flow cytometry) (**A**). The cells with low autophagosome production are located at L1 and the cells with high autophagosome production are at L2. Percentage of cells located at L2 (**B**). Data are expressed as mean ± SEM (*n* = 3). The ANOVA test was performed to compare the groups of cells located at L2, *p* < 0.0001 in both cell lines. The differences between the means of the groups and the control group were analyzed by Dunnett’s post-hoc test. The significant differences were expressed as * *p* <0.05 and *** *p* <0.001.

In a context-dependent manner, autophagy maintains a dynamic interplay between cytoprotection and cytostasis during cancer progression. In recent years, researchers have focused on blocking autophagic pathways to control cancer spread and identify the critical role autophagy plays in cell survival and cell death [30]. Figure 8A shows histograms representative of the fluorescence intensity of an autophagosome marker in cells SW480 and SW620. Cells with the lowest production of autophagosomes (lower autophagy) are located at L1, and cells with the highest production of autophagosomes (higher autophagy) are at L2. Figure 8B shows the percentage of cells located at L2. There, cells treated with 0.1 µM rapamycin (used as a positive control) have the highest percentage (greater autophagy). Likewise, Figure 8B indicates that SW480 and SW620 cells treated with 30 mg LMPE/mL for 48 h decreased by 20% and 12%, respectively, compared to controls (*p <* 0.001). That is, treatment with LMPE causes a decrease in autophagy in SW480 and SW620 cells.

### 3.9. Detection of MMP-7 and MMP-9 Expression

**Figure 9:**
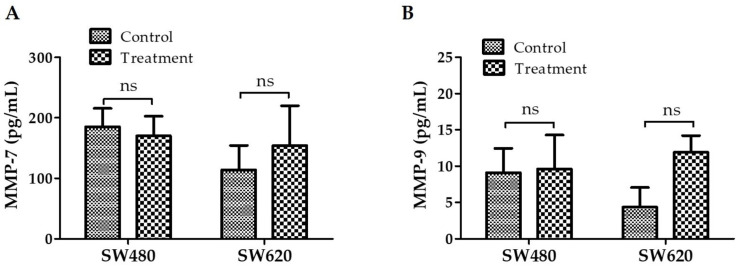
Effect of LMPE on MMP-7 (**A**) and MMP-9 (**B**) expression in SW480 and SW620 cell lines. Cells were exposed to 30 mg LMPE/mL for 48 h. Untreated cells (0 mg LMPE/mL) were used as a control. Data are expressed as mean ± SEM (*n* = 3). The differences between the control and the treated cells were analyzed with the Student’s *t* test, ns = non-significant difference.

In addition to being crucial for preserving extracellular homeostasis, MMPs can significantly contribute to the invasion and spread of cancer cells. MMPs are, therefore, attractive therapeutic targets [17]. Figure 9A shows that there were no significant differences in MMP-7 expression between cells treated with 30 mg LMPE/mL for 48 h and control cells in both cell lines, —SW480 (*p* = 0.6898) and SW620 (*p* = 0.5115)—. Figure 9B shows that there were also no significant differences in MMP-9 expression between cells treated with 30 mg LMPE/mL for 48 h and control cells in both cell lines, —SW480 (*p* = 0.9128) and SW620 (*p* = 0.0666)—. That is, treatment with EPLM did not modulate the expression of MMP-7 and MMP-9 in SW480 and SW620 cells.

### 3.10. Invasion Assay via ECM

**Figure 10:**
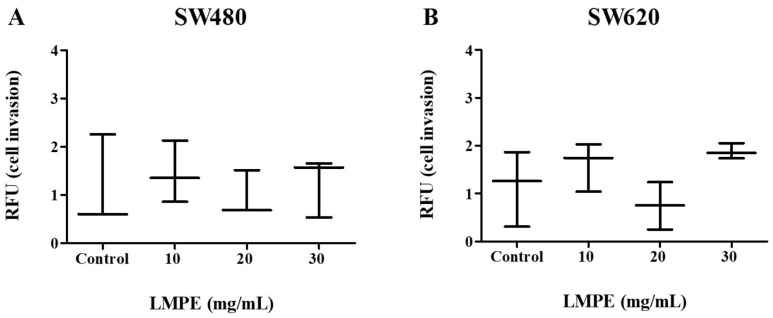
Effect of LMPE on invasion through an extracellular matrix in SW480 (**A**) and SW620 (**B**) cells. Cells were exposed to different treatment concentrations (10, 20, and 30 mg LMPE/mL) for 18 h. Untreated cells (0 mg LMPE/mL) were used as a control. Data were expressed as median (interquartile range) (*n* = 3). The Kruskal-Wallis test was performed to compare the groups *p* = 0.8569 (SW480) and *p* = 0.1230 (SW620).

An essential step in tumor metastasis is invasion through the ECM [31]. An assay based on the Boyden chamber principle was used to evaluate the effect of LMPE on invasion through the ECM in SW480 and SW620 cells. Figure 10 shows that there were no significant differences in invasion through an ECM in SW480—*p* = 0.8569 (Figure 10A)—and SW620—*p* = 0.1230 (Figure 10B)—cells treated with 10, 20, 30 mg LMPE/mL for 18 h and control cells (0 mg LMPE/mL). These results, together with those presented in Figure 9, suggest that LMPE treatment has no effect on invasion processes in these CRC cells.

## 4. Discussion

This research demonstrated, for the first time, that an aqueous extract of lyophilized mango pulp (LMPE) induces apoptosis without mitochondrial membrane permeabilization mediation and decreases autophagy in human adenocarcinoma cells (SW480) and their metastatic-derived cells (SW620).

A key strategy in the chemoprevention of CRC is to promote the consumption of functional foods with bioactive compounds capable of blocking, delaying, or reversing the carcinogenesis process [3]. The mango fruit is widely consumed worldwide [4] and is considered a functional food for its high content of bioactive compounds such as carotenoids, polyphenols, and fiber [32].

Previously, our group had evaluated the cytotoxic effect of LMPE on SW480 and SW620 cells, using skin fibroblasts (non-malignant cells) as a control. The IC_50_ values of LMPE at 48 h of exposure for SW480, SW620, and skin fibroblasts were 43, 29, and 129 mg/mL, respectively. This demonstrated that LMPE was cytotoxic and selective for SW480 and SW620 cell lines with respect to non-malignant skin fibroblasts. It was also found that at concentrations below 50 mg LMPE/mL at 48 h of exposure, no cytotoxic effect was observed in skin fibroblasts (non-malignant cells). As possible mechanisms of action of LMPE, we suggest increased intracellular ROS production, cell cycle arrest in the G2/M phase, and, probably, induction of apoptosis. In that study, the combined annexin V-fluorescein isothiocyanate (FITC)/propidium iodide (PI) staining allowed the identification of apoptotic cells [11].

In the present study, increased DNA fragmentation and modulation of proteins associated with apoptosis signaling pathways were observed in SW480 and SW620 cells exposed to LMPE. When taken together, these results confirm the apoptotic activity of LMPE on these CRC cells. Different studies have demonstrated the ability of some bioactive compounds present in mango to promote apoptosis in cancer cells [33,34,35], specifically in the context of CRC, Noratto et al. showed the apoptotic effect of polyphenolic mango extracts (10 mg gallic acid equivalent/mL and 24 h exposure) on SW480 cells [9] and Lauricella et al. reported an increase in oxidative stress associated with DNA fragmentation and apoptosis in CRC Caco-2 and HCT116 cell lines treated with an ethanolic mango peel extract [8].

The two main apoptotic signaling pathways are extrinsic and intrinsic (mitochondrial). The mitochondrial membrane potential was measured to evaluate mitochondrial mediation in possible apoptotic pathways modulated by LMPE in SW480 and SW620 cells. The results showed that treatment with LMPE did not generate mitochondrial membrane permeabilization, which is required to promote apoptosis intrinsically [36].

To further advance the study of apoptotic pathways, flow cytometries, and immunoassays were performed and allowed us to observe the effect of the treatment on the modulation of proteins related to apoptosis. 

In the SW480 and SW620 cells exposed to LMPE, an increase in the expression of the antiapoptotic protein Bcl-2 was evidenced, which decreases oligomerization and pore-forming activity in the mitochondria by pro-apoptotic proteins such as Bax and BAK [14]. Therefore, this increase in Bcl-2 expression could explain why no mitochondrial membrane permeabilization was observed in cells treated with the LMPE. These Bcl-2 expression results are consistent with the hypothesis that apoptosis in the SW480 and SW620 cell lines occurs through a pathway that does not require mitochondrial membrane permeabilization mediation, as discussed above.

Specifically, in SW480 cells exposed to LMPE, the analysis of proteins related to apoptosis revealed overexpression of death receptors DR4, Fas, and TNF-R1, which are involved in the extrinsic apoptotic pathway [37]. Likewise, the expression of cleaved caspase-3 increased, which favors biological processes such as DNA fragmentation [36] and the apoptosis observed in this cell line. When taken together from the observed results, it is proposed that the pathway activated by the effect of LMPE on SW480 cells is the extrinsic pathway without mitochondrial membrane permeabilization mediation. As mentioned above, apoptotic death without mitochondrial membrane permeabilization was observed in SW620 cells; however, the down-regulation of cleaved caspase-3 by the LMPE effect raises doubts that the activated pathway is the extrinsic pathway; therefore, it was not possible to propose a specific apoptotic pathway as the mechanism of LMPE-induced death in SW620 cells. It has been shown that SW480 and SW620 cell lines are different when it comes to susceptibility to apoptosis induction by pathways involving death receptors. Hewitt et al. showed that SW480 was susceptible to cell death by the anti-Fas monoclonal CH11, but SW620 was not [19]. Likewise, it is reported that SW480 cells are sensitive to TRAIL, while their SW620-derived metastatic cells are resistant [20]. This could partly explain the observed results. It is important to highlight that LMPE contains a mixture of bioactive compounds that can generate cellular stress in SW480 and SW620 cells through various signaling pathways. Furthermore, modulation of apoptosis-related proteins by LMPE does not necessarily imply their involvement in apoptotic pathways. Therefore, future studies using apoptotic pathway-specific ligands or blockers are required to better understand the signaling pathways involved in the chemopreventive ability of LMPE against SW480 and SW620 cells.

The process of autophagy involves the sequestration of cellular components, such as macro proteins or even entire organelles, into lysosomes for degradation [38]. This type of cell death depends mechanically on autophagic machinery and manifests itself with an extensive production of autophagic vesicles and the consequent lysosomal degradation [36]. Currently, the role of autophagy is part of the research on the prevention and treatment of CRC [39]. Some studies suggest that autophagy prevents the onset of the tumor and the first steps of its progression. However, they also suggest that once the tumor is established, inhibition of autophagy may induce cancers with low aggressiveness [40]. The findings of the present investigation showed that LMPE caused a decrease in autophagosome production (autophagy) in SW480 and SW620 cells. It is documented that autophagy proves to be protective for malignant cells against oxidative stress conditions and DNA fragmentation [41], such as those generated by LMPE in SW480 and SW620 cells. It is also reported that reducing autophagy is a particularly useful strategy against cancer cells with RAS mutations, as is the case of SW480 and SW620 cells [42]. Therefore, the decrease in autophagy could sensitize these cells to treatment with LMPE.

Regulation of autophagy and apoptosis overlap when the BH3 domain of the Beclin 1 protein (autophagy-inducing protein) interacts with antiapoptotic proteins of the Bcl-2 family, including Bcl-2, which causes autophagy inhibition [43]. Thus, the increase in Bcl-2 expression observed in LMPE-treated SW480 and SW620 cells could partly explain the reduction in autophagosome production. It is important to consider that LMPE is a source of various bioactive compounds, which can act through different targets of action (pleiotropic), thus increasing its effectiveness [44] on SW480 colon cancer cell lines and their SW620 metastatic-derived cells.

This is the first work to explore the relationship between mango or mango extracts and autophagy in CRC cells. However, it is documented how other natural products can modulate autophagy. For example, in HCT116 colon cancer cells, apigenin modulated the PI3K/Akt/mTOR signaling pathway to induce autophagy [45]. Recently, combining anticancer drugs with autophagy blockers has been proposed, but the efficacy of this approach is still under debate [46].

The SW480 colon cancer cell lines and their SW620 metastatic-derived cells are a suitable model to investigate the progression of colorectal cancer [19]. Therefore, it was studied whether LMPE could have effects on the expression of MMP-7 and MMP-9 and on the invasion processes through an ECM in these CRC cells. MMPs are members of the zinc-dependent endopeptidase family. Collagen and other crucial ECM proteins are broken down by these proteases. Specifically, it has been reported that MMP-7 contributes to tumor invasion, proliferation, and antiapoptotic potential in CRC, while MMP-9 has been implicated in extracellular protein proteolysis during tumor invasion in CRC [47]. Although there are no previous reports on the effect of mango or its extracts on cell invasion processes in CRC cells, it has been documented that lycopene, a bioactive substance found in mango, has been shown to have chemopreventive properties by inhibiting MMP-7 production and cell invasion in human colon cancer HT-29 cells [48]. The results of the assays showed that the LMPE did not modulate the expression of MMP-7 and MMP-9, nor did it have an effect on invasion through an ECM in the SW480 and SW620 cell lines.

## 5. Conclusions

LMPE is a source of bioactive compounds that generates DNA fragmentation and apoptosis without mitochondrial membrane permeabilization in SW480 and SW620 cells. Additionally, it decreases autophagy in these cell lines, which could sensitize them to damage to the DNA generated by LMPE. The LMPE did not modulate the expression of matrix metalloproteinases 7 and 9, nor did it affect cellular invasion processes in the SW480 colon cancer cell lines and their SW620 metastatic-derived cells.

## Figures and Tables

**Table 1:** Biological processes and KEGG pathways after the STRING^®^ analysis of LMPE-modulated proteins for SW480 and SW620 cells. Analysis of protein interactions.

SW480		SW620	
**Biological Process**	**FRD**	**Biological Process**	**FRD**
Neuronal apoptotic process	2.41 × 10^−12^	Apoptotic process	1.34 × 10^−7^
Regulation of apoptotic processes	2.41 × 10^−12^	Negative regulation of apoptotic processes	1.34 × 10^−7^
Programmed cell death	2.70 × 10^−12^	Regulation of apoptotic processes	1.36 × 10^−7^
**via KEGG**	**FRD**	**via KEGG**	**FRD**
Apoptosis	2.89 × 10^−21^	Apoptosis—multiple species	1.42 × 10^−11^
Apoptosis—multiple species	1.14 × 10^−20^	Apoptosis	2.17 × 10^−10^
Platinum Drug Resistance	1.44 × 10^−17^	Small-Cell Lung Carcinoma	3.7 × 10^−7^

FDR: False Discovery Rate; KEGG: Kyoto Gene and Genome Encyclopedia.

## Data Availability

Not applicable.
